# Association Between *GSDMB* Gene Polymorphism and Cervical Cancer in the Northeast Chinese Han Population

**DOI:** 10.3389/fgene.2022.860727

**Published:** 2022-06-27

**Authors:** Songxue Li, Xiaoying Li, Shuang Zhang, Yanan Feng, Tianshuang Jia, Manning Zhu, Lei Fang, Liping Gong, Shuang Dong, Xianchao Kong, Zhenzhen Wang, Litao Sun

**Affiliations:** ^1^ Cancer Center, Department of Ultrasound Medicine, Zhejiang Provincial People’s Hospital, Hangzhou, China; ^2^ Department of Ultrasound, The 2nd Affiliated Hospital of Harbin Medical University, Harbin, China; ^3^ Department of Obstetrics and Gynecology, The 2nd Affiliated Hospital of Harbin Medical University, Harbin, China

**Keywords:** cervical cancer, cervical squamous intraepithelial lesion, pyrocytosis, GSDMB, SNP

## Abstract

**Objective:** The purpose of this study was to investigate the relationship between *GSDMB* gene polymorphism and genetic susceptibility to cervical cancer in the Han population in Northeast China.

**Methods:** In this case–control study, the genotypes and alleles of rs8067378 in the *GSDMB* gene were analyzed by multiplex polymerase chain reaction (PCR) and next-generation sequencing methods in 482 cervical cancer (CC) patients, 775 cervical squamous intraepithelial lesion (SIL) patients, and 495 healthy women. The potential relationships between the SNP of the *GSDMB* gene with SIL and CC were analyzed by multivariate logistic regression analysis combined with 10,000 permutation tests.

**Results:** In the comparison between the SIL group and the control group, the genotype and allele distribution frequencies of rs8067378 SNP of the *GSDMB* gene were statistically significant (*p* = 0.0493 and *p* = 0.0202, respectively). The allele distribution frequencies of rs8067378 were also statistically significant in the comparison between high-grade cervical squamous intraepithelial lesion (HSIL) and low-grade cervical squamous intraepithelial lesion (LSIL) groups with control group ( *p* = 0.0483 and *p* = 0.0330, respectively). Logistic regression analysis showed that after adjusting for age, the rs8067378 SNP of the *GSDMB* gene was significantly associated with the reduced risk of SIL under the dominant model (*p* = 0.0213, OR = 0.764, CI = 0.607–0.961) and the additive model (*p* = 0.0199, OR = 0.814, and CI = 0.684–0.968), and its mutant gene G may play a role in the progression of healthy people to LSIL and even HSIL as a protective factor. However, there was no significant association between cervical cancer and its subtypes with the control group (*p* > 0.05). After 10,000 permutations, there was still no correlation that has provided evidence for the accuracy of our study.

**Conclusion:** The results of this study showed that rs8067378 single nucleotide polymorphism of the *GSDMB* gene may reduce the risk of SIL and protect the susceptibility to cervical precancerous lesions in the Northeast Chinese Han population, but it has no significant correlation with the progression of cervical cancer.

## 1 Introduction

Cervical cancer is the fourth most frequently diagnosed cancer and the fourth leading cause of cancer death in women. It is estimated that by 2020, there were 604,000 new cases and 342,000 deaths in the world (Sung et al., 2021). The progression of cervical cancer is a complex process with multiple steps and factors. In addition to the risk factors such as long-term chronic inflammation caused by high-risk human papillomavirus (HPV) persistent infection, long-term oral contraceptives, and fertility and sexual life disorder, the occurrence of cervical cancer also has obvious individual genetic susceptibility (Nkfusai et al., 2019; Olusola et al., 2019; Sadri Nahand et al., 2020; van der Waal et al., 2020; Yang et al., 2020). Single nucleotide polymorphism (SNP) is the most common form of genetic variation, which widely exists in the human genome and is related to the susceptibility to a variety of cancers such as cervical cancer. Therefore, the study of SNP will also contribute to the early diagnosis and treatment of cervical cancer (Shastry, 2009; Tan, 2017).

Gasdermin B (GSDMB) is a member of the GSDM protein superfamily that exists only in vertebrates. The *GSDMB* gene, located on chromosome 17q21.2, has a conserved gasdermin domain, a potential nuclear localization signal, and two nuclear receptor-binding motifs LXX(L/I)L. It is 1518 bp length and encodes 411 amino acids. It is often expressed in normal esophageal and gastrointestinal epithelium and bronchial epithelium of asthmatic lungs (Sun et al., 2008; Zheng et al., 2020). GSDMB has six splice variants, and GSDMB-1 is the longest and most important subtype which is expressed in human cancer cell lines (Feng et al., 2018).

Recently, several studies have identified that the expression of GSDMB has a potential relationship with the occurrence and development of tumors. GSDMB can regulate its lipid binding and pore-forming activity through different mechanisms of the intramolecular domain and then participate in pyrocytosis (Chen et al., 2019; Li et al., 2020). Pyrocytosis is a pro-inflammatory form that regulates cell death. Its essence is a cascade amplification of inflammatory response. It often participates in the body’s immune defense and plays a “double-edged sword” role in the occurrence and development of tumor (Li et al., 2020). In addition, the level of sulfatide on the apical surface of many tumor epithelial cells is significantly increased, and the metastatic ability of tumor cells is related to the specific binding of GSDMB to sulfatide (Chao et al., 2017). Increasingly, a large number of case–control studies have also suggested that the *GSDMB* gene polymorphism is associated with susceptibility to many autoimmune diseases and malignant tumors such as cervical cancer (Hergueta-Redondo et al., 2016; Wiemels et al., 2018; Molina-Crespo et al., 2019; Li et al., 2020; Li et al., 2021). At present, the relationship between *GSDMB* gene polymorphism and the risk of cervical cancer in the Han population in Northeast China has not been reported. Therefore, this study aimed to explore the relationship between rs8067378 polymorphism of the *GSDMB* gene and the risk of cervical cancer in the Northeast Chinese Han population. Since cervical squamous intraepithelial lesions, as a form of precancerous lesions, reflect the continuous development of cervical cancer (de Rycke et al., 2020), we will also study whether rs8067378 polymorphism is associated with cervical squamous intraepithelial lesions in Han women in Northeast China.

## 2 Materials and Methods

### 2.1 Study Subjects

#### 2.1.1 Case Group

All subjects in this case–control study were recruited from the same center (Department of Obstetrics and Gynecology, Second Affiliated Hospital of Harbin Medical University, Harbin City, Heilongjiang Province, China) from September 2014 to October 2018. The case group included 482 cases of primary cervical cancer without any treatment, among which were 416 cases of SCC, 36 cases of AUC, and 30 cases of other pathological types. Another 775 patients with SIL were recruited, which included 619 patients with HSIL and 156 patients with LSIL. All the patients were confirmed by pathology experts of the Second Affiliated Hospital of Harbin Medical University. Exclusion criteria: cervical benign lesions, cervical benign tumors, other cervical malignant tumors, cervical lesions after preoperative chemoradiotherapy, and patients combined with other cancers.

#### 2.1.2 Control Group

The control group consisted of 496 healthy people from the physical examination center of the same hospital in the same period. Inclusion criteria: there was no abnormality in the ThinPrep cytologic test (TCT), no cancer, or family history of cancer. Exclusion criteria: all gynecological diseases and surgical history of gynecological diseases, hypo-immunity and immune diseases, cervical surgery, skin or genital condyloma, and other cancer history, etc.

All subjects signed informed consent, and all studies in this report were approved by the Ethics Committee of the Second Affiliated Hospital of Harbin Medical University.

### 2.2 Candidate SNP Selection

Based on the data from the previous literature and combined with the characteristics of the East Asian population in the dbSNP database, the SNP rs8067378 (A > G) at 17q12 identified by previous cervical cancer GWAS (Shi et al., 2013) was selected for the current study.

### 2.3 Extraction and Genotyping of DNA

After fasting for 12 h, 1 ml peripheral venous blood samples were collected and put into a 2% EDTA-Na₂ anticoagulant tube. The samples were numbered and stored in an −80°C refrigerator until DNA extraction. The genomic DNA of all subjects was extracted from peripheral venous blood samples for genotyping according to the standard steps of the TIANamp Genomic DNA Kit (Tiangen Biotech, Beijing, China). All genotyping experiments for the selected SNP (rs8067378) were performed using two rounds of multiplex PCR combined with next-generation sequencing methods by the Shanghai Biowing Applied Biotechnology Company (Chen et al., 2016). Primer3 online software (v0.4.0) was used to amplify the primer sequences. PCR was performed for SNP analysis with the following primers: rs8067378 (sense, 5′-GTT​GAC​AGT​CAA​AAC​AAA​AAC​CTG-3′). After two rounds of multiplex PCR, the PCR products were mixed into the centrifugal tube, then sealed with the sealing membrane, and placed overnight. The mixture was purified using a TIANgel Midi Purification Kit (Tiangen Biotech, China). The purified PCR products were then paired-end sequenced (2 × 150 bp) by the Illumina HiSeq XTen platform according to the manufacturer’s instructions. The read data were compared to the human reference genome using the Burrows–Wheeler (BWA, v0.7.12) (Slater et al., 2009), and SAMtools (v0.1.19) (Li, 2011) was used for SNP calling and genotyping. In total, ninety-one samples were randomly selected for blind DNA replication to control the quality of genotyping.

### 2.4 Physiological and Biochemical Index Collection and Analysis

The general information and clinical data of each subject in the case group were recorded in detail: age, menstrual history, smoking and drinking history, past disease history, operation history, family history of tumor, relevant laboratory examination results, histopathological diagnosis, clinically confirmed diagnosis, and relevant gynecological examination (ultrasound or colposcopy), etc.

In addition, the general situation and some clinical data of the healthy control population in the physical examination center during the same period were recorded: age, ThinPrep cytologic test (TCT), and HPV, etc. Moreover, due to the influence of traditional Chinese culture, the subjects in this study almost refused to answer questions about sexual life history, so this part of the data could not be collected.

Squamous cell carcinoma (SCC) antigen, carcino-embryonic antigen (CEA), alpha-fetoprotein (AFP), CA125, D-dimer, and other biochemical indicators were detected by the laboratory of the Second Affiliated Hospital of Harbin Medical University. The histopathological diagnosis of cervical tissue samples was confirmed by the pathology department of the Second Affiliated Hospital of Harbin Medical University.

### 2.5 Allele, Genotypes, and Statistics

SPSS 21.0 software was used to analyze the clinical characteristics of the case group and the control group. Student’s *t*-test was used to compare the continuous variables among the groups. The Pearson chi-square test was used to compare the categorical variables between groups (the Fisher exact test was used when the expected value was less than 5). Plink1.9 single nucleotide polymorphism software was used for genotyping analysis. The Hardy–Weinberg equilibrium was analyzed. We established the following three models: dominant model: major allele homozygous vs. heterozygous + minor allele homozygous; recessive model: major allele homozygous + heterozygous vs. minor allele homozygous; and additive model: major allele homozygous vs. heterozygous vs. minor allele homozygous. The 95% confidence interval (CI) and odds ratio (OR) of each genetic model were calculated. Multivariate logistic regression analysis was performed to adjust age to evaluate the association of SNP with cervical cancer, cervical intraepithelial neoplasia, and their subtypes. Moreover, the chi-square test combined with logistic regression analysis was used to analyze the relationship between genotypes and alleles with clinical parameters. All statistical analyses showed that *p* < 0.05 had statistical significance.

## 3 Results

### 3.1 SNP Genotype and Quality Controls

The SNP of the *GSDMB* gene was rs8067378, and its genotype distributions in the CC and SIL groups conform to the Hardy–Weinberg equilibrium (*p* = 0.9108 and *p* = 0.9508, respectively). In addition, we controlled the quality of 73 samples in this experiment, and the accuracy rate is 98.5%, and the error rate is within a reasonable range, which ensures the reliability and repeatability of the follow-up research results.

### 3.2 Clinical Characteristics of the Study Population

The clinical characteristics of all CC patients, SIL patients, and healthy controls in this study were statistically analyzed, as shown in Table 1. The results showed that compared with the healthy control group, the average age of CC and SIL patients was old, and the comparison between the control group and CC and SIL groups was statistically significant (*p* < 0.0001). The analysis of other clinical characteristics of the CC group and SIL group showed that there were significant differences in menarche age and amenorrhea between the two groups (*p* < 0.05). In addition, smoking was significantly correlated between the two groups (*p* = 0.0091) while drinking was not (*p* = 0.4952).

**TABLE 1:** Clinical characteristics in the control group with the CC and SIL groups.

	Control	CC	SIL	P1	P2	P3
Age (years)	39.44 ± 8.22	49.33 ± 9.29	42.22 ± 9.68	**<0.0001**	**<0.0001**	**<0.0001**
Menarche age (years)	--	14.92 ± 1.86	14.61 ± 1.90	--	--	**0.0058**
Amenorrhea n (%)	--	216 (44.8%)	148 (19.1%)	--	--	**<0.0001**
Smoking n (%)	--	30 (6.22%)	23 (2.97%)	--	--	**0.0091**
Drinking n (%)	--	4 (0.83%)	4 (0.52%)	--	--	0.4952

CC, cervical cancer; SIL, cervical squamous intraepithelial lesion.

*p*-value (P1, comparison of controls with CC; P2, comparison of controls with SIL; P3, comparison of CC with SIL).

*p* values < 0.05 are considered statistically significant, and shown in bold.

### 3.3 Analysis of Genotypes and Allele Distribution Frequencies

The genotypes and allele distribution frequencies of *GSDMB* gene polymorphism (rs8067378) in the CC group and its subgroups with the healthy control group are shown in Table 2. The results of the chi-square test showed that the genotypes and allele frequencies of rs8067378 single nucleotide polymorphism of the *GSDMB* gene had no significant difference between the CC group and healthy control group (*p* > 0.05), and there was no significant difference between the SCC and AUC groups with healthy control group (*p* > 0.05). Logistic regression analysis showed that after adjusting for age, rs8067378 single nucleotide polymorphism was not significantly associated with cervical cancer and its subtypes (*p* > 0.05). After 10,000 permutations, the results were still not statistically significant. The consistency of 10,000 permutations further supports the reliability of our results.

**TABLE 2:** Comparison of genotypes and allele distribution frequencies between the CC group and its subgroups with the healthy control group.

Genotype and allele	Control	Case	P	OR [95% CI]	Statistical model	P′	P′′	OR [95% CI]
	Controls (n= 495)	CC(n = 482)						
AA	259 (52.3%)	235 (48.8%)			Dominant	0.2054	0.2095	0.829[0.620–1.108]
AG	189 (38.2%)	199 (41.3%)			Recessive	0.5305	0.5327	0.852[0.516–1.406]
GG	47 (9.49%)	48 (9.96%)	0.5322		Additive	0.2132	0.2088	0.868[0.696–1.084]
A	707 (71.4%)	669 (69.4%)						
G	283 (28.6%)	295 (30.6%)	0.3290	0.908[0.747–1.102]				
	Controls (n= 495)	SCC (n = 416)						
AA	259 (52.3%)	204 (49.0%)			Dominant	0.2967	0.2968	0.851[0.628–1.152]
AG	189 (38.2%)	169 (40.6%)			Recessive	0.4040	0.4110	0.801[0.476–1.348]
GG	47 (9.49%)	43 (10.3%)	0.6110		Additive	0.2443	0.2437	0.872[0.692–1.098]
A	707 (71.4%)	577 (69.4%)						
G	283 (28.6%)	255 (30.6%)	0.3362	1.104[0.902–1.351]				
	Controls (n= 495)	AUC(n = 36)						
AA	259 (52.3%)	16 (44.4%)			Dominant	0.2542	0.2631	0.659[0.321–1.350]
AG	189 (38.2%)	16 (44.4%)			Recessive	0.5203	0.5204	0.680[0.209–2.207]
GG	47 (9.49%)	4 (11.1%)	0.6589		Additive	0.2441	0.2462	0.727[0.425–1.243]
A	707 (71.4%)	48 (66.7%)						
G	283 (28.6%)	24 (33.3%)	0.3909	0.801[0.481–1.332]				

CC, cervical cancer; SCC, squamous carcinoma of cervical; AUC, adenocarcinoma of the uterine cervical; OR, odds ratio; CI, confidence interval.

P: *p*-value (control vs. CC; control vs. SCC; control vs. AUC ); *p*-values <0.05 are shown in bold.

P’: age was adjusted.

P”: the *p*-value was calculated using 10,000 permutations for each model to correct the multiple tests.

Table 3 shows the comparison of genotypes and allele distribution frequencies between the SIL group and its subgroups with healthy control group. The results of the chi-square test showed that the genotype frequencies of rs8067378 single nucleotide polymorphism of the *GSDMB* gene were statistically significant between the SIL group and healthy control group (*p* = 0.0493), and the A/G allele frequency was also statistically significant between the two groups (*p* = 0.0202). Compared with the healthy control group (28.6%), the G mutation of rs8067378 accounted for 32.5% in the HSIL group and 34.9% in the LSIL group, and the A/G allele frequency was significantly correlated between the HSIL group and LSIL group with the healthy control group (*p* = 0.0483 and 0.0330, respectively). Logistic regression analysis showed that after adjusting for age, rs8067378 SNP was statistically significant between the SIL group and its subgroup (HSIL group) with the healthy control group in the dominant model and additive model (*p* = 0.0213 and 0.0356 in the dominant model, and *p* = 0.0199 and 0.0406 in the additive model, respectively). The difference between the LSIL group and healthy control group was statistically significant in the additive model (*p* = 0.0308). It can be seen that under the additive model, rs8067378 plays a protective role in the progression of healthy people to LSIL and even HSIL (*P*2 = 0.0308, OR = 0.740, CI = 0.564–0.973; *P*1 = 0.0406, OR = 0.827, CI = 0.689–0.992). This correlation still exists after 10,000 permutations.

**TABLE 3:** Comparison of genotypes and allele distribution frequencies between the SIL group and its subgroups with the healthy control group.

Genotype and allele	Control	Case	P	OR [95% CI]	Statistical model	P′	P′′	OR [95% CI]
	Control (n= 495)	SIL (n = 775)						
AA	259 (52.3%)	351 (45.3%)			Dominant	**0.0213**	**0.0228**	0.764[0.607–0.961]
AG	189 (38.2%)	337 (43.5%)			Recessive	0.1861	0.1881	0.773[0.528–1.132]
GG	47 (9.49%)	87 (11.2%)	**0.0493**		Additive	**0.0199**	**0.0208**	0.814[0.684–0.968]
A	707 (71.4%)	1,039 (67.0%)						
G	283 (28.6%)	511 (33.0%)	**0.0202**	0.814[0.684–0.968]				
	Control (n= 495)	HSIL (n = 619)						
AA	259 (52.3%)	283 (45.7%)			Dominant	**0.0356**	**0.0366**	0.773[0.608–0.983]
AG	189 (38.2%)	270 (43.6%)			Recessive	0.3067	0.3102	0.811[0.543–1.211]
GG	47 (9.49%)	66 (10.7%)	0.0903		Additive	**0.0406**	**0.0401**	0.827[0.689–0.992]
A	707 (71.4%)	836 (67.5%)						
G	283 (28.6%)	402 (32.5%)	**0.0483**	0.832[0.694–0.999]				
	Control (n= 495)	LSIL (n = 156)						
AA	259 (52.3%)	68 (43.6%)			Dominant	0.0631	0.0633	0.702[0.484–1.019]
AG	189 (38.2%)	67 (42.9%)			Recessive	0.0928	0.0916	0.614[0.348–1.084]
GG	47 (9.49%)	21 (13.5%)	0.1180		Additive	**0.0308**	**0.0327**	0.740[0.564–0.973]
A	707 (71.4%)	203 (65.1%)						
G	283 (28.6%)	109 (34.9%)	**0.0330**	0.745[0.569–0.977]				

SIL, cervical squamous intraepithelial lesion; HSIL, high-grade cervical squamous intraepithelial lesion; LSIL, low-grade cervical squamous intraepithelial lesion; OR, odds ratio; CI, confidence interval.

P: *p*-value (control vs. SIL; control vs. HSIL; control vs. LSIL ); *p* values <0.05 are considered statistically significant, and shown in bold.

P’: age was adjusted.

P”: the *p*-value was calculated using 10,000 permutations for each model to correct the multiple tests.

Table 4 shows the comparison of genotypes and allele distribution frequencies of rs8067378 SNP of the *GSDMB* gene between the CC group and SIL group. The results show that there is no significant difference in genotypes and allele distribution frequencies of rs8067378 SNP between the CC group and SIL group (*p* > 0.05). Logistic regression analysis showed that there was no significant difference in rs8067378 SNP between the CC group and SIL group after adjusting for age (*p* > 0.05). After 10,000 permutations, the results were still not statistically significant.

**TABLE 4:** Comparison of genotypes and allele distribution frequencies between the CC group and the SIL group.

Genotype and allele	CC (n = 482)	SIL (n = 775)	P	OR [95% CI]	Statistical model	P′	P′′	OR [95% CI]
AA	235 (48.8%)	351 (45.3%)			Dominant	0.2191	0.2195	1.165[0.913–1.485]
AG	199 (41.3%)	337 (43.5%)			Recessive	0.5878	0.5914	1.116[0.751–1.657]
GG	48 (9.96%)	87 (11.2%)	0.4616		Additive	0.2419	0.2423	1.115[0.929–1.339]
A	669 (69.4%)	1,039 (67.0%)						
G	295 (30.6%)	511 (33.0%)	0.2165	1.115[0.938–1.326]				

CC, cervical cancer; SIL, cervical squamous intraepithelial lesion; OR, odds ratio; CI, confidence interval.

P: *p*-value (CC vs. SIL ); *p*-values <0.05 are shown in bold.

P’: age was adjusted.

P”: the *p*-value was calculated using 10,000 permutations for each model to correct the multiple tests.

**FIGURE 1:**
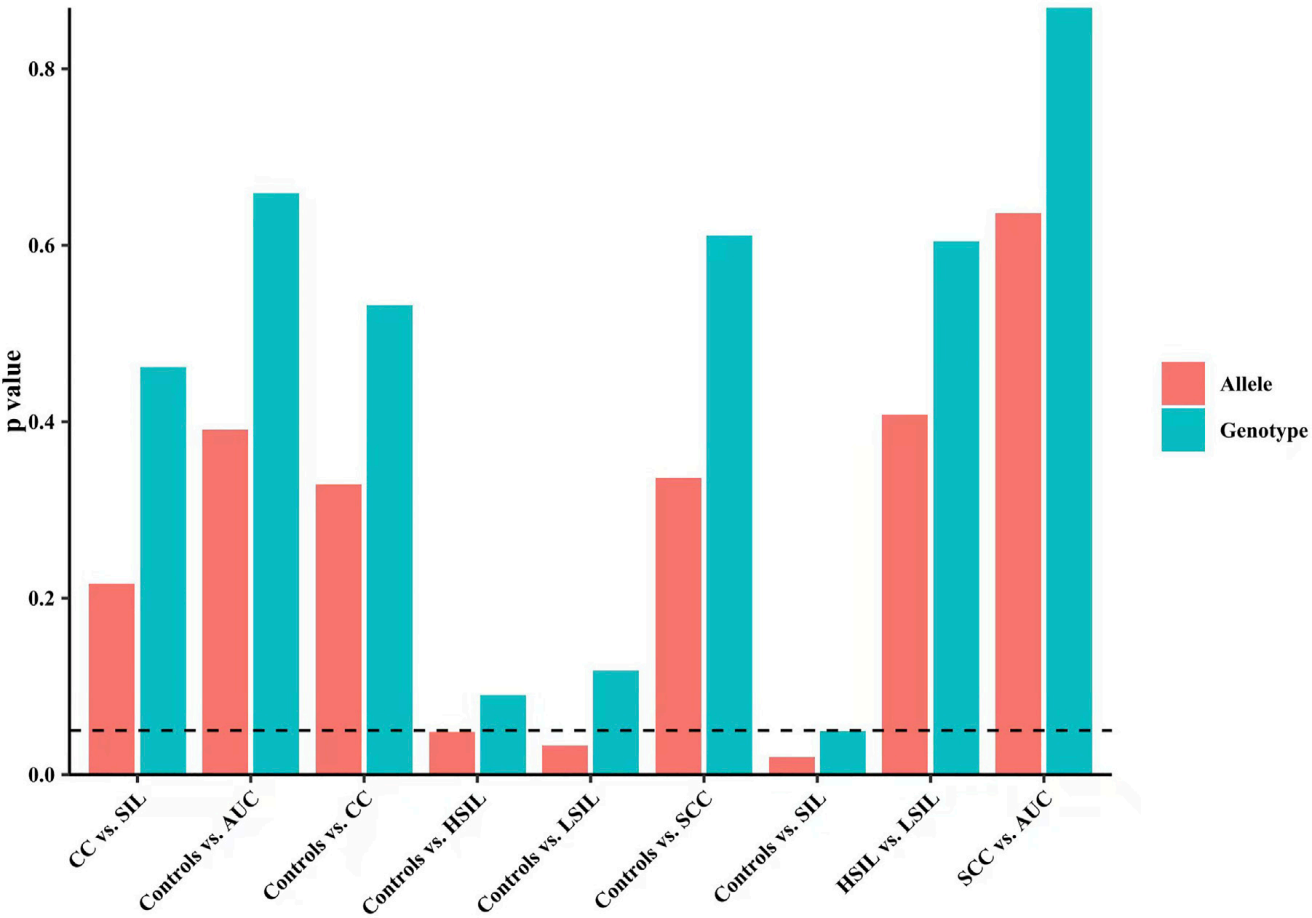
Results of chi-square tests show the comparison of genotypes and allele distribution frequencies between groups.

**FIGURE 2:**
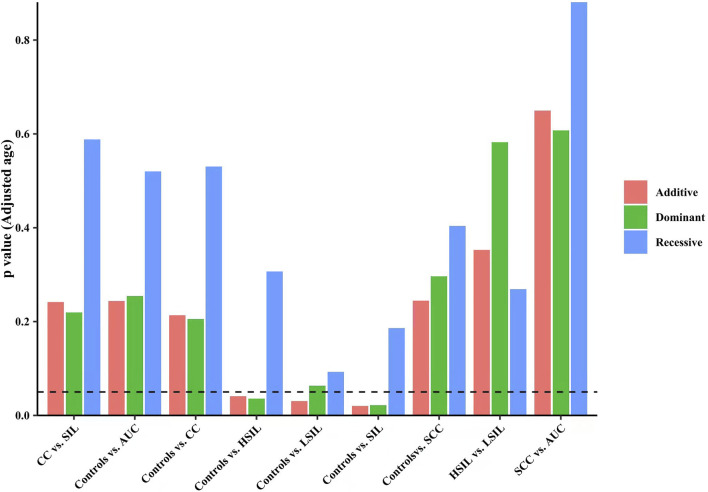
Results of the logistic regression analysis after adjusting for age show the comparison of genotypes and allele distribution frequencies between groups.

**FIGURE 3:**
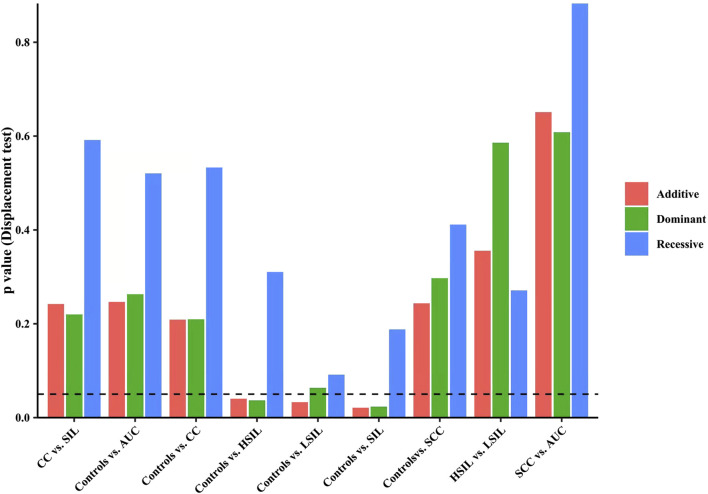
Results after 10,000 permutation tests show the comparison of genotypes and allele distribution frequencies between groups.

Finally, we made intra-subgroup comparisons. Tables 5, 6 summarized the comparisons of genotypes and allele distribution frequencies of rs8067378 SNP of the *GSDMB* gene between the SCC group and the AUC group, and the HSIL group and the LSIL group, respectively. The results show that there is no significant difference in genotypes and allele distribution frequencies of rs8067378 SNP between the two intra-subgroups (*p* > 0.05). After adjusting for age, there is still no significant difference among the genetic models (*p* > 0.05). After 10,000 permutations, the results were still not statistically significant, which has provided evidence for the accuracy of our study. In addition, in order to more clearly and definitely show the comparison of genotypes and allele distribution frequencies between groups, we drew three graphs: Figure 1 shows the results of chi-square tests, Figure 2 shows the results of logistic regression analysis after adjusting for age, and Figure 3 shows the results after 10,000 permutation tests.

**TABLE 5:** Comparison of genotypes and allele distribution frequencies between the SCC group and AUC group.

Genotype and allele	SCC (n = 416)	AUC (n = 36)	P	OR [95% CI]	Statistical model	P′	P′′	OR [95% CI]
AA	204 (49.0%)	16 (44.4%)			Dominant	0.6073	0.6082	1.197[0.603–2.374]
AG	169 (40.6%)	16 (44.4%)			Recessive	0.8808	0.8827	1.087[0.367–3.222]
GG	43 (10.3%)	4 (11.1%)	0.8690		Additive	0.6495	0.6511	1.123[0.680–1.856]
A	577 (69.4%)	48 (66.7%)						
G	255 (30.6%)	24 (33.3%)	0.6362	0.884[0.530–1.474]				

SCC, squamous carcinoma of cervical; AUC, adenocarcinoma of the uterine cervical; OR, odds ratio; CI, confidence interval.

P: *p*-value (SCC vs. AUC ); *p*-values <0.05 are shown in bold.

P’: age was adjusted.

P”: the *p*-value was calculated using 10,000 permutations for each model to correct the multiple tests.

**TABLE 6:** Comparison of genotypes and allele distribution frequencies between the HSIL group and LSIL group.

Genotype and allele	HSIL (n = 619)	LSIL (n = 156)	P	OR [95% CI]	Statistical model	P′	P′′	OR [95% CI]
AA	283(45.7%)	68 (43.6%)			Dominant	0.5822	0.5860	1.105[0.774–1.578]
AG	270(43.6%)	67 (42.9%)			Recessive	0.2686	0.2713	1.347[0.794–2.286]
GG	66(10.7%)	21 (13.5%)	0.6043		Additive	0.3525	0.3554	1.132[0.872–1.470]
A	836(67.5%)	203(65.1%)						
G	402(32.5%)	109(34.9%)	0.4080	0.896[0.690–1.163]				

HSIL, high-grade cervical squamous intraepithelial lesion; LSIL, low-grade cervical squamous intraepithelial lesion; OR, odds ratio; CI, confidence interval.

P: *p*-value (HSIL, vs. LSIL ); *p*-values <0.05 are shown in bold.

P’: age was adjusted.

P”: the *p*-value was calculated using 10,000 permutations for each model to correct the multiple tests.

### 3.4 Analysis of Genotypes and Allele Frequencies With Clinical Parameters

In the CC group and SIL group, the comparisons of genotypes and allele distribution frequencies of *GSDMB* gene rs806778 polymorphism with clinical parameters are shown in Tables 7–10. It is worth noting that in patients with cervical cancer, allele frequencies are significantly correlated with amenorrhea (*p* = 0.0328). After logistic regression analysis, the correlations still exist (*p* = 0.0330). In addition, no significant correlation was observed between genotypes and allele frequencies with other clinical parameters (*p* > 0.05). Moreover, there was also no significant correlation between genotypes and allele frequencies with clinical parameters in patients with cervical squamous intraepithelial lesions (*p* > 0.05).

**TABLE 7:** Comparison of genotype distribution frequencies with clinical parameters in the CC group.

Parameter	ALL	AA	AG	GG	P		P′	OR [95% CI]
Histology					0.8204			
SCC	416	198	175	43		AG/AA	0.6203	0.834 [0.406–1.712]
AUC	36	19	14	3		GG/AA	0.6206	0.727 [0.206–2.567]
Menarche age					0.7900			
<15 years	229	113	91	25		AG/AA	0.5695	1.119 [0.760–1.646]
≥15 years	234	111	100	23		GG/AA	0.8370	0.937 [0.502–1.748]
Amenorrhea					0.1083			
Yes	220	96	97	27		AG/AA	0.1336	0.744 [0.506–1.095]
No	246	129	97	20		GG/AA	0.0663	0.551 [0.292–1.041]
Parity					0.2469			
Never	29	10	16	3		AG/AA	0.1002	0.504 [0.222–1.141]
Ever	396	196	158	42		GG/AA	0.6207	0.714 [0.188–2.708]
**HPV**					0.7248			
( - )	8	5	3	0		AG/AA	0.8795	1.123 [0.250–5.044]
( + )	83	46	31	6		GG/AA	0.9740	78185.319
D-dimer					0.8988			
≤243 ng/ml	392	188	163	41		AG/AA	0.8218	1.086 [0.531–2.217]
>243 ng/ml	36	17	16	3		GG/AA	0.7446	0.809 [0.227–2.890]
SCC					0.4132			
≤1.5 ng/ml	205	99	89	17		AG/AA	0.6246	0.907 [0.613–1.342]
>1.5 ng/ml	245	119	97	29		GG/AA	0.2951	1.419 [0.737–2.733]
CEA					0.6549			
≤5 ng/ml	183	81	80	22		AG/AA	0.8885	0.945 [0.428–2.085]
>5 ng/ml	31	15	14	2		GG/AA	0.3679	0.491 [0.104–2.310]
CA125					0.3928			
≤35 U/ml	197	88	86	23		AG/AA	0.5395	1.302 [0.560–3.028]
>35 U/ml	26	11	14	1		GG/AA	0.3238	0.348 [0.043–2.835]
**CA199**					0.0898			
≤37 U/ml	194	83	87	24		AG/AA	0.1570	0.477 [0.171–1.330]
>37 U/ml	18	12	6	0		GG/AA	0.9654	0.000

P: p-value; p-values <0.05 are shown in bold.

P’: logistic regression analysis.

**TABLE 8:** Comparison of allele distribution frequencies with clinical parameters in the CC group.

Parameter	ALL	A	G	P	P′	OR [95% CI]
Histology				0.5275	0.5280	0.841[0.492–1.438]
SCC	832	571	261			
AUC	72	52	20			
Menarche age				0.8926	0.8926	1.019[0.772–1.347]
<15 years	458	317	141			
≥15 years	468	322	146			
Amenorrhea				**0.0328**	**0.0330**	0.739[0.559–0.976]
Yes	440	289	151			
No	492	355	137			
Parity				0.2413	0.2428	0.720[0.415–1.250]
Never	58	36	22			
Ever	792	550	242			
HPV				0.5295	0.5320	1.515[0.412–5.573]
( - )	16	13	3			
( + )	166	123	43			
D-dimer				0.9031	0.9036	0.968[0.573–1.634]
≤243 ng/ml	784	539	245			
>243 ng/ml	72	50	22			
SCC				0.5975	0.5981	1.079[0.812–1.435]
≤1.5 ng/ml	410	287	123			
>1.5 ng/ml	490	335	155			
CEA				0.4535	0.4542	0.798[0.443–1.440]
≤5 ng/ml	366	242	124			
>5 ng/ml	62	44	18			
CA125				0.6940	0.6941	0.882[0.472–1.648]
≤35 U/ml	394	262	132			
>35 U/ml	52	36	16			
CA199				**0.0272**	**0.0328**	0.375[0.152–0.923]
≤37 U/ml	388	253	135			
>37 U/ml	36	30	6			

P: *p*-value; *p* values <0.05 are considered statistically significant, and shown in bold.

P’: logistic regression analysis.

**TABLE 9:** Comparison of genotype distribution frequencies with clinical parameters in the SIL group.

Parameter	ALL	AA	AG	GG	P		P′	OR [95% CI]
Histology					0.4017			
HSIL	619	285	269	65		AG/AA	0.6491	0.916 [0.628–1.336]
LSIL	156	66	68	22		GG/AA	0.1781	0.684 [0.394–1.189]
Menarche age					0.1808			
<15 years	445	201	186	58		AG/AA	0.6156	1.081 [0.798–1.463]
≥15 years	325	148	148	29		GG/AA	0.1244	0.679 [0.414–1.113]
Amenorrhea					0.4376			
Yes	156	68	74	14		AG/AA	0.3993	0.853 [0.589–1.235]
No	607	278	258	71		GG/AA	0.5038	1.240 [0.660–2.332]
Parity					0.5740			
Never	57	29	21	7		AG/AA	0.3058	1.357 [0.756–2.436]
Ever	654	293	288	73		GG/AA	0.9427	1.032 [0.435–2.450]
HPV					0.4522			
( - )	27	10	15	2		AG/AA	0.3170	0.656 [0.288–1.497]
( + )	440	195	192	53		GG/AA	0.6978	1.359 [0.289–6.391]
D-dimer					0.6396			
≤243 ng/ml	743	336	323	84		AG/AA	0.8382	0.892 [0.296–2.681]
>243 ng/ml	16	7	6	3		GG/AA	0.4418	1.714 [0.434–6.770]
SCC					0.5749			
≤1.5 ng/ml	472	211	208	53		AG/AA	0.7599	1.092 [0.620–1.926]
>1.5 ng/ml	58	26	28	4		GG/AA	0.3802	0.612 [0.205–1.831]
CEA					0.7043			
≤5 ng/ml	191	87	84	20		AG/AA	0.9663	1.036 [0.203–5.276]
>5 ng/ml	6	3	3	0		GG/AA	0.9719	0.000
CA125					0.3617			
≤35 U/ml	193	90	82	21		AG/AA	0.3162	1.585 [0.644–3.903]
>35 U/ml	23	9	13	1		GG/AA	0.4929	0.476 [0.057–3.967]
CA199					0.9019			
≤37 U/ml	177	80	79	18		AG/AA	0.8067	0.868 [0.279–2.697]
>37 U/ml	15	7	6	2		GG/AA	0.7765	1.270 [0.243–6.630]

P: *p*-value; *p*-values <0.05 are shown in bold.

P’: logistic regression analysis.

**TABLE 10:** Comparison of allele distribution frequencies with clinical parameters in the SIL group.

Parameter	ALL	A	G	P	P′	OR [95% CI]
Histology				0.2180	0.2183	0.849[0.655–1.102]
HSIL	1,238	839	399			
LSIL	312	200	112			
Menarche age				0.3557	0.3558	0.903[0.728–1.121]
<15 years	890	588	302			
≥15 years	650	444	206			
Amenorrhea				0.9314	0.9315	1.012[0.776–1.319]
Yes	312	210	102			
No	1,214	814	400			
Parity				0.5893	0.5895	1.121[0.741–1.696]
Never	114	79	35			
Ever	1,308	874	434			
HPV				0.8422	0.8422	0.943[0.530–1.677]
( - )	54	35	19			
( + )	880	582	298			
D-dimer				0.5960	0.5965	1.216[0.590–2.508]
≤243 ng/ml	1,486	995	491			
>243 ng/ml	32	20	12			
SCC				0.6301	0.6302	0.903[0.596–1.369]
≤1.5 ng/ml	944	630	314			
>1.5 ng/ml	116	80	36			
CEA				0.5861	0.5884	0.694[0.185–2.607]
≤5 ng/ml	382	258	124			
>5 ng/ml	12	9	3			
CA125				0.9470	0.9468	1.022[0.532–1.963]
≤35 U/ml	386	262	124			
>35 U/ml	46	31	15			
CA199				0.9242	0.9238	1.039[0.471–2.292]
≤37 U/ml	354	239	115			
>37 U/ml	30	20	10			

P: *p*-value; *p*-values <0.05 are shown in bold.

P’: logistic regression analysis.

## 4 Discussion

Cervical cancer is characterized by a high incidence rate and high mortality rate, and it is a major public problem affecting the health of middle-aged women, especially in countries with low resources (Hu et al., 2018; Arbyn et al., 2020; Zhang et al., 2020). Persistent infection of high-risk HPV is the most important risk factor associated with cervical cancer (Faridi et al., 2011; Paaso et al., 2019). However, studies have shown that other risk factors associated with HPV may also play important roles in the pathogenesis of cervical cancer, such as immune or genetic factors. SNP has become an important biomarker for locating cancer, such as cervical cancer (Shastry, 2009). Therefore, this case–control study was conducted to research the association between *GSDMB* gene polymorphism and the risk of cervical cancer in the Han population in Northeast China. In this study, we investigated the relationship between rs8067378 SNP of the *GSDMB* gene and susceptibility to cervical cancer in Han population in Northeast China. According to the histopathological classification, CC patients were further divided into the SCC group and AUC group, and the SIL group was divided into the HSIL group and LSIL group. Then, intragroup and intergroup comparisons were performed.

Our results replicated previous studies and showed that the average age of the CC group was 49.33 ± 9.29 years, and the average age of the SIL group was 42.22 ± 9.68 years. This is consistent with the conclusion that the peak of CC in the Chinese mainland is 40–60 years old and the peak of SIL is 30–50 years old (Wang et al., 2018; Liu et al., 2020). This may be related to the fact that women in the 30–40 age group are in the period of sexual activity and childbearing, while most of the women after the age of 50 are postmenopausal (Misra et al., 2018).

In this case–control study, we investigated the relationship between rs8067378 polymorphism of the *GSDMB* gene and the risk of CC in the Han population in Northeast China. The results showed that there was no significant correlation between rs8067378 polymorphism with CC and its subtypes. These are different from the existing research studies. A genome-wide association study on cervical cancer in the Chinese Han population proved that rs8067378 SNP is a susceptible site for cervical cancer (Shi et al., 2013). Miura et al. (2016) showed for the first time that there was a significant correlation between rs8067378 SNP and invasive cervical cancer and further proved that Japanese women with the GG genotype were at high risk of invasive cervical cancer. Lutkowska et al. (2017) studied the relationship between rs8067378 SNP and stage III and IV of cervical cancer in the Polish population, in which the G allele plays a role in the diffusion of tumor cells to adjacent tissues, indicating that rs8067378 SNP increases the risk of occurrence and development of cervical cancer. Such findings have also been confirmed in some parts of southern China. Based on previous studies, the specific mechanism of GSDMB in the occurrence and development of cervical cancer is not clear. Some studies suppose that GSDMB may participate in the regulation of the estrogen–estrogen receptor-target gene expression pathway under the action of endogenous and exogenous estrogen (Sun et al., 2008). Studies have shown that *GSDMB* containing two nuclear receptor-binding motifs can be used as a nuclear receptor co-activator, recruited in the estrogen receptor to form a complex, provide related enzymatic activity and scaffolding function, promote the high expression of E6/E7 oncogene in patients with high-risk HPV persistent infection, and cause uncontrolled cell proliferation, cervical intraepithelial lesions, and even cervical cancer (Sun et al., 2008). In addition, these differences may be related to the sample size and case determination protocol. Also, the genetic background and lifestyle may also change the genetic susceptibility of the *GSDMB* gene, which explain the differences in the intensity of research correlation between different regions and races. Therefore, we should repeat the study with a larger sample, to determine the relationship between *GSDMB* gene polymorphism with SCC and AUC and even other special types of CC.

GSDMB can participate in pyrocytosis and the production of inflammatory factors, and studies found that GSDMB can be highly expressed in cervical inflammatory lesions and precancerous lesions (Sun et al., 2008). Therefore, in order to further study the role of *GSDMB* gene rs8067378 polymorphism in the development of cervical cancer in healthy women, we also analyzed the susceptibility of SIL. Surprisingly, under the dominant and additive models, the rs8067378 polymorphism of the *GSDMB* gene is significantly correlated with SIL and its subtypes, and rs8067378 single nucleotide polymorphism may reduce the risk of SIL and protect the susceptibility to SIL. This may be related to the dual role of GSDMB-mediated pyrocytosis in tumor, and the occurrence of pyrocytosis is concentration-dependent on GSDMB. Studies have pointed out that (Kayagaki et al., 2015; Tsuchiya, 2020) macrophages and monocytes lacking GSDMB almost completely lose the transmission ability of lipopolysaccharide (LPS) in the cytoplasm and will not activate inflammatory caspases, so they will not induce pyrocytosis. In the process of pyrocytosis, caspases are the inducer and GSDMB is the substrate. Precursor caspases are activated by a variety of inflammatory bodies such as the NOD-like receptor (NLR) and are absent in melanoma 2 (AIM2). Active caspases bind to the cutting site of GSDMB. This process releases the N-terminal and C-terminal, which are inserted into the cell membrane to cause pyrocytosis and release a large number of inflammatory factors such as tumor necrosis factor (TNF), interleukin-1β (IL-1β), and interferon-α (IFN-α). This process can not only directly kill pathological and tumor cells but also further recruit immune cells, so as to expand the inflammatory response. Therefore, some scholars believe that active caspases cutting GSDMB-induced pyrocytosis is a positive feedback mechanism in the immune process, so that most newborn tumor cells are eliminated by the process of pyrocytosis before the formation of tumor. It plays a role in preventing the further development of precancerous lesions (Dinarello, 2009; Guo et al., 2015; Xia et al., 2019; Zhou and Fang, 2019; Ruan et al., 2020). This is consistent with the research results of Zhang WH. The study has shown that after CC cells are infected by HPV, AIM2 can play a tumor inhibitory role by inducing pyrocytosis (So et al., 2018).

The study confirmed for the first time that *GSDMB* gene polymorphism was significantly correlated with cervical squamous intraepithelial lesions, but not with cervical cancer in the Han population in Northeast China. The studies of Miura et al. and Lutkowska et al. have confirmed that *GSDMB* gene polymorphism was significantly associated with cervical cancer. Compared with previous existing studies, the sample size in our study was relatively large, including 482 cases of cervical cancer, 775 cases of cervical squamous intraepithelial lesions, and 495 healthy controls. Moreover, our study was analyzed from multiple perspectives such as cervical cancer subgroups and cervical precancerous lesions. A large sample size is very important for exploring and verifying the potential mechanism of cervical cancer and precancerous lesions in Chinese women and will also play an important role in the research of different pathological types of cervical cancer and precancerous lesions in the future. In addition, we also added the comparison of genotypes and alleles with clinical parameters to understand more clinically related risk factors. However, this study also has some limitations. First, this study adopts a case–control study, which inevitably has selective bias. Second, our study population is limited to Han women in Northeast China, so it is necessary to conduct a multicenter and large-scale replication study to confirm the results, including people from different regions and races. Finally, cervical cancer is a complex disease affected by multiple genes. This study only discussed the limited loci of the *GSDMB* gene, and other functional genes and loci have not been studied. Therefore, the combined role of the *GSDMB* gene and other genes in the occurrence and development of cervical cancer needs to be further explored.

In conclusion, our study is the first to prove that rs8067378 polymorphism of the *GSDMB* gene is significantly associated with SIL in the Northeast Chinese Han population. Rs8067378 polymorphism may be used as a protective factor to reduce the risk of SIL and even CC in the Han population in Northeast China during the progression of healthy people to LSIL and even HSIL. It is necessary to conduct more in-depth research to understand the specific mechanism of the *GSDMB* gene in the occurrence and development of cervical cancer, so as to provide theoretical support for the diagnosis and treatment of cervical cancer in the future.

## Data Availability

The datasets presented in this study can be found in online repositories. The names of the repository/repositories and accession number(s) can be found at: https://doi.org/10.6084/m9.figshare.19351877.
