# Corrigendum: Association between *GSDMB* gene polymorphism and cervical cancer in the Northeast Chinese Han population

**DOI:** 10.3389/fgene.2023.1298000

**Published:** 2023-11-13

**Authors:** Songxue Li, Xiaoying Li, Shuang Zhang, Yanan Feng, Tianshuang Jia, Manning Zhu, Lei Fang, Liping Gong, Shuang Dong, Xianchao Kong, Zhenzhen Wang, Litao Sun

**Affiliations:** ^1^ Cancer Center, Department of Ultrasound Medicine, Zhejiang Provincial People’s Hospital, Hangzhou, China; ^2^ Department of Ultrasound, The 2nd Affiliated Hospital of Harbin Medical University, Harbin, China; ^3^ Department of Obstetrics and Gynecology, The 2nd Affiliated Hospital of Harbin Medical University, Harbin, China

**Keywords:** cervical cancer, cervical squamous intraepithelial lesion, pyrocytosis, GSDMB, SNP

In the published article, there was an error in the **article type**. Instead of “Systematic Review,” it should be “Original Research.”

The authors apologize for this error and state that this does not change the scientific conclusions of the article in any way. The original article has been updated.

